# Clinical outcomes of new multifocal intraocular lenses with hydroxyethyl methacrylate and comparative results of contrast sensitivity, objective scatter, and subjective photic phenomena

**DOI:** 10.1186/s12886-022-02600-x

**Published:** 2022-09-21

**Authors:** Yong Woo Lee, Chul Young Choi, Kun Moon, Yong Jin Jeong, Sang Il An, Je Myung Lee, Jong Ho Lee, Min Cheol Seong

**Affiliations:** 1 Department of Ophthalmology, Kangwon National University Hospital, Kangwon National University School of Medicine, Chuncheon, Republic of Korea; 2 Hanyang University School of Medicine, Seoul, Republic of Korea; 3 Seoulbalgeunsesang Eye Clinic, Seoul, Republic of Korea; 4 Department of Ophthalmology, Kangbuk Samsung Hospital, Sungkyunkwan University School of Medicine, Seoul, Republic of Korea; 5 Department of Ophthalmology, Hanyang University Guri Hospital, 153, Gyeongchun-ro, Guri-si, Gyeonggi-do 11923 Republic of Korea

**Keywords:** Hydroxyethyl methacrylate, Multifocal intraocular lens, Contrast sensitivity

## Abstract

**Background:**

We investigate the performance of new hydrophobic diffractive multifocal intraocular lenses (IOL) with hydroxyethyl methacrylate (HEMA) and compare their optical quality, contrast sensitivity, and subjective photic phenomena.

**Methods:**

Medical records of patients who underwent routine simple cataract surgery and insertion of an existing multifocal IOL (TFNT, TF group) or a new multifocal IOL (CNWT, CN group) were retrospectively reviewed. Clinical data was collected 2 months postoperatively and included optical quality analysis system (OQAS) indices, contrast sensitivity, and subjective degrees of photic phenomena.

**Results:**

One hundred thirty-five eyes of 135 patients were included (CN group, 71; TF group, 64). There was no significant difference between the two groups in the visual acuity and defocus curve. The indices of OQAS did not show a significant difference between groups. Contrast sensitivity was significantly better in the CN group at all degrees, including the area under the log contrast sensitivity function (*p* = 0.01). The subjective photic phenomena survey showed better results for the CN group, with the proportion of patients reporting no photic phenomena as 9.9% and 3.1% in the CN and TF groups, respectively. The proportion of patients who reported severe photic phenomena was 11.3% in the CN group and 25.0% in the TF group. Although the follow-up period was only 2 months, glistening, surface scattering, and posterior capsule opacity were not observed in any patient.

**Conclusions:**

The new multifocal IOL with HEMA is safe, and provides stable visual acuity as well as superior contrast sensitivity and lower subjective photic phenomena, over the prior IOL.

## Background

Currently, cataract surgery is gradually developing into presbyopia surgery, which corrects refractive errors and near vision, as well as provides treatment for the removal of opacity in the crystalline lens. In particular, with the development of a diffractive multifocal intraocular lens (IOL), presbyopia-corrective IOLs have made a breakthrough. The diffractive IOL, which started as a bifocal lens, has recently been developed into a trifocal or quadrifocal IOL, providing not only improved distance and near vision, but also intermediate vision, enhancing quality of life for many people [1]. The first quadrifocal diffractive IOL TFNT (Acrysof Panoptix, Alcon, Fort Worth, Texas, USA) is a C-loop, 1-piece hydrophobic acrylic IOL based on SN60WF (Acrysof IQ, Alcon, Fort Worth, Texas, USA), which has been widely used as a monofocal IOL for cataract corrections. It provides stable distance, intermediate, and near vision [2]. However, owing to the optical characteristics of multifocal IOLs, there have been limitations in terms of glare and contrast sensitivity [3]. Especially for lenses based on Acrysof materials, long-term glistening and surface scattering can be strongly influenced by multifocal, rather than monofocal lenses [4–8].

Recently, release of CNA0T0 (Clareon, Alcon, Fort Worth, Texas, USA) has provided an IOL that replaced phenylethyl methacrylate (PEMA) with hydroxyethyl methacrylate (HEMA) by improving the material technology that the manufacturer had previously maintained [9, 10]. Using these advantages, a diffractive multifocal IOL CNWT (Clareon Panoptix, Alcon, Fort Worth, Texas, USA), which utilizes novel materials based on existing multifocal IOL optics, has become commercially available. We present the clinical outcomes of a multifocal IOL comprised of novel materials, including HEMA, and compare the vision quality, contrast sensitivity, and subjective degree of photic phenomena against an existing multifocal IOL.

## Methods

### Subjects

This was a retrospective medical record survey. From December 2021 to April 2022, the medical records of all patients undergoing CNWT (CN group) and TFNT (TF group) IOL implantation during cataract surgery performed at the Seoulbalgeunsesang Eye Clinic were reviewed. This study was approved by the Institutional Review Board of the Hanyang University Guri Hospital and the need for written informed consent was waived by the Institutional Review Board of the Hanyang University Guri Hospital due to retrospective nature of the study. We complied with the Declaration of Helsinki.

For statistical comparisons, only the right eye was included from the patients who underwent bilateral surgery. Patients over 65 years of age or with organic abnormalities other than simple cataracts, including those of the cornea and retina, were excluded. Patients with posterior capsule ruptures or radial tears during surgery or those with posterior capsule opacity under mydriatic slit-lamp examination were also excluded. Patients with severe cataracts whose visual acuity could not be corrected before surgery were also excluded for the sake of the accuracy of the results. High astigmatism that could not be corrected with the provided toric IOL was excluded. Post operative refractive errors over 0.5D of spherical equivalent or astigmatism were recused due to the possibility of affected visual acuity or photic phenomena.

### Intraocular lenses

From December 2021 to early January of 2022, TFNT was selected for all cataract patients. In mid-January of 2022, as CNWT became commercially available, it was inserted to all the patients. In all patients, the IOL power closest to the emmetropia calculated by the Barrett Universal II formula, with a provided constant (lens factor = 1.94) using the IOLMaster® 700 (Carl Zeiss Meditec Inc., Jena, Germany), was inserted, and for corneal astigmatism, the closest astigmatism correction power was also inserted on the correct axis.

### Surgical technique

Cataract surgery was performed by an experienced ophthalmologist under topical anesthesia. When using toric IOL, the correct axis was marked on the slit lamp before surgery. 5.0 mm capsulorhexis was performed using a femtosecond laser (Catalys laser system, Abbott Medical Optics, Inc., Santa Ana, Ca, USA), followed by injection of viscosurgical device, phacoemulsification through a temporal clear or limbal corneal incision (2.8 mm size), after which an IOL was inserted.

### Patient examinations

All patients underwent slit-lamp examination, tonometry, pupil size (Nidek ARK-1, Nidek Co., Ltd., Aichi, Japan), mydriasis fundus examination, and IOLMaster® 700 for biometric parameters before surgery.

**Fig. 1:**
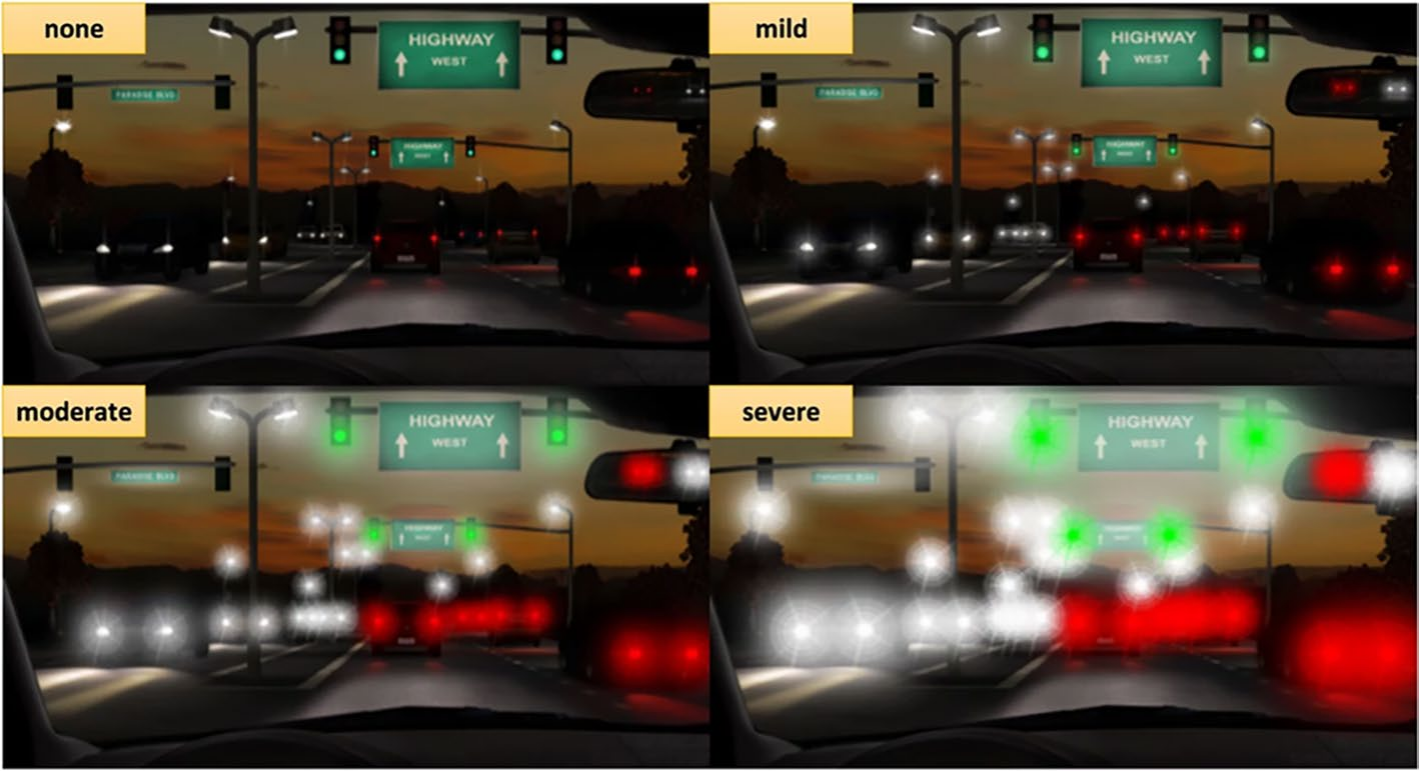
Photic phenomena images using the glare and halo simulator were provided to the patients for subjective photic phenomena survey

Follow-up was scheduled at 1 day, 1 week, 1 month, and 2 months after surgery. The manifest refraction test, uncorrected distance visual acuity (UDVA) with Snellen standard chart (Smart LC-13, Medizs Co., Ltd. Korea) at 6 m under photopic condition (85 cd/m^2^), defocus curve, uncorrected optical quality analysis system index (OQAS, HD Analyzer II, Visometrics SL, Terrassa, Spain), contrast sensitivity test (CGT-2000, Takagi Co Ltd., Nagano-Ken, Japan), and subjective photic phenomena surveys were performed in all patients 2 months after surgery. The uncorrected binocular and monocular defocus curves were obtained from 0D to − 4.00D in 0.5D steps with randomization under photopic conditions (85 cd/m^2^). In the OQAS test, the objective scatter index (OSI), modulation transfer function (MTF) cutoff value, and Strehl ratio (SR) values ​​were collected. Contrast sensitivity tests were performed under mesopic conditions (0.6 cd/m^2^) at 6.3°, 4.0°, 2.5°, 1.6°, 1.0°, and 0.64° at a distance of 5 m according to manufacturer’s recommendation, and the contrast sensitivity value and area under the log contrast sensitivity function (AULCSF) values were collected. In the subjective photic phenomena survey, photic phenomena simulation images (Fig. 1) were presented to patients, and they were asked to determine the degree among four options: none, mild, moderate, and severe [11].

### Statistical analysis

SPSS version 25.0 (IBM, Armonk, New York, USA) was used for statistical comparisons of each group. Normality of the data samples was assessed using the Kolmogorov-Smirnov test. Independent t-tests for numeric variables and Chi square tests for categorical variables were utilized for statistical comparisons. McNemar test was used to compare photic phenomena survey. Significance was set at *p* < 0.05. Linear regression analysis was performed to determine the correlation between OQAS indicators and contrast sensitivity.

## Results

A total of 135 patients (135 eyes) were included in the study. Among them, 71 eyes were included in the CN group, and 64 in the TF group. The patients included in the binocular defocus measurement were 68 in the TF group and 60 in the CN group. The average age was 58.06 ± 5.03 years in the CN group and 57.67 ± 4.54 years in the TF group (*p* = 0.64). There were 49 (69.0%) and 44 (68.8%) women in the CN and TF groups, respectively; this difference in sex was not statistically significant (*p* = 0.97).

**Fig. 2:**
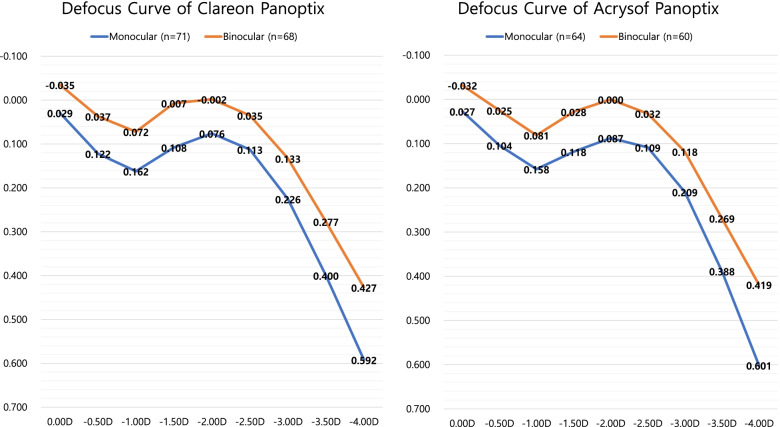
Photopic uncorrected (refractive error within 0.5D) LogMAR monocular and binocular defocus curves of multifocal intraocular lenses with Clareon Panoptix and Acrysof Panoptix. There were no statistically significant differences between the two groups in all diopters. D diopters; LogMAR logarithm of the minimal angle of resolution

CDVA (corrected distance visual acuity), cataract stage, axial length, anterior chamber depth, and corneal refractive index, which were measured before surgery, as well as the IOL power did not show any statistically significant differences between the groups (Table 1). The results of the defocus curve of the binocular visual acuity of the eyes showed a visual acuity of 20/20 or better in 0D and decreased from − 0.5D to − 1.5D. Mean binocular visual acuity was 20/20 or better at − 2.0D and decreased again. Mean binocular visual acuity of 16/20 or better was recorded from 0D to − 2.5D. Figure 2 shows monocular and binocular uncorrected visual acuity defocus curves. In both groups, there was no significant difference in all diopters in both monocular and binocular visual acuity. The monocular logarithm of the minimum angle of resolution UDVA of the CN group was 0.029 ± 0.050 and the TF group was 0.027 ± 0.065, indicating no statistically significant difference (Table 2).

**Table 1:** Comparison of preoperative ocular parameters and cataract stages between the Clareon Panoptix and Acrysof Panoptix groups

	Clareon Panoptix Group	Acrysof Panoptix Group	*p* Value
Eyes (n)	71	64	
Age (years)	58.06 ± 5.03 [50, 65]	57.67 ± 4.54 [41, 65]	0.64
Female (n)	49 (69.0%)	44 (68.8%)	0.97
LOCS III grade			
Cortical	2.63 ± 1.29 [0, 5]	2.81 ± 1.42 [0, 6]	0.45
Nuclear	3.55 ± 0.94 [0, 5]	3.41 ± 1.11 [0, 5]	0.42
Posterior subcapsular	0.44 ± 0.97 [0, 4]	0.50 ± 0.98 [0, 3]	0.71
LogMAR CDVA	0.34 ± 0.22 [0.15, 1.00]	0.35 ± 0.27 [0.15, 1.00]	0.80
Pupil size (mm)	5.37 ± 0.74 [3.2, 6.8]	5.22 ± 0.82 [3.1, 6.9]	0.25
Axial length (mm)	24.42 ± 1.51 [21.82, 27.17]	24.13 ± 1.34 [22.38, 29.07]	0.24
ACD (mm)	3.29 ± 0.37 [2.50, 4.16]	3.25 ± 0.31 [2.49, 4.39]	0.51
Average K (D)	43.75 ± 1.57 [41.22, 46.62]	43.86 ± 1.90 [41.51, 46.78]	0.70
K astigmatism (D)	0.81 ± 0.53 [0.00, 3.02]	0.83 ± 0.59 [0.00, 2.82]	0.86
IOL power (D)	18.51 ± 4.52 [7.5, 26.5]	19.48 ± 3.15 [9.5, 24.5]	0.16
IOL cylindrical power (D)	0.92 ± 0.83 [0.00, 3.75]	0.96 ± 0.91 [0.00, 3.75]	0.74

Values are presented as mean ± standard deviation. [minimum, maximum]

*LOCS* lens opacities classification system, *LogMAR* logarithm of minimal angle of resolution, *CDVA* corrected distance visual acuity, *ACD* anterior chamber depth, *K* keratometry, IOL intraocular lens

**Table 2:** Comparison of visual acuity, optical quality analysis and contrast sensitivity between the Clareon Panoptix and Acrysof Panoptix groups

	Clareon Panoptix (*n* = 71)	Acrysof Panoptix (*n* = 64)	*p* value
LogMAR UDVA	0.029 ± 0.050	0.027 ± 0.065	0.75
SE (D)	−0.026 ± 0.273	−0.027 ± 0.246	0.98
Refractive cylinder (D)	0.368 ± 0.107	0.353 ± 0.122	0.68
OQAS			
OSI	1.594 ± 0.868	1.856 ± 1.138	0.13
MTF cut-off value (CPD)	30.723 ± 11.191	29.193 ± 10.719	0.42
SR	0.147 ± 0.060	0.138 ± 0.055	0.38
Contrast sensitivity			
AULCSF	1.449 ± 0.168	1.358 ± 0.233	0.01
6.3°	0.019 ± 0.010	0.026 ± 0.026	0.04
4.0°	0.020 ± 0.009	0.030 ± 0.037	0.03
2.5°	0.026 ± 0.013	0.037 ± 0.035	0.02
1.6°	0.045 ± 0.020	0.059 ± 0.045	0.02
1.0°	0.078 ± 0.035	0.105 ± 0.079	0.01
0.64°	0.156 ± 0.074	0.201 ± 0.126	0.01

Values are presented as mean ± standard deviation

*LogMAR* logarithm of minimal angle of resolution, *UDVA* uncorrected distance visual acuity, *SE* spherical equivalent, *D* diopters, *OQAS* optical quality analysis system, *OSI* objective scattering index, *MTF* modulation transfer function, *CPD* cycle per degree, *SR* Strehl ratio, *AULCSF* area under the log contrast sensitivity function

In the OQAS test, mean OSI value was lower in the CN group; but the difference was not statistically significant. The MTF cutoff value and SR did not show a significant difference either. However, in the contrast sensitivity test, the CN group showed significantly better results than the TF group at all degrees, and the AULCSF was 1.449 ± 0.168 in the CN group, significantly higher than 1.358 ± 0.233 in the TF group (*p* = 0.01) (Table 2). As a result of linear regression analysis, only OSI among OQAS indicators showed a significant correlation with AULCSF (R^2^ = 0.098, *p* < 0.001).

**Fig. 3:**
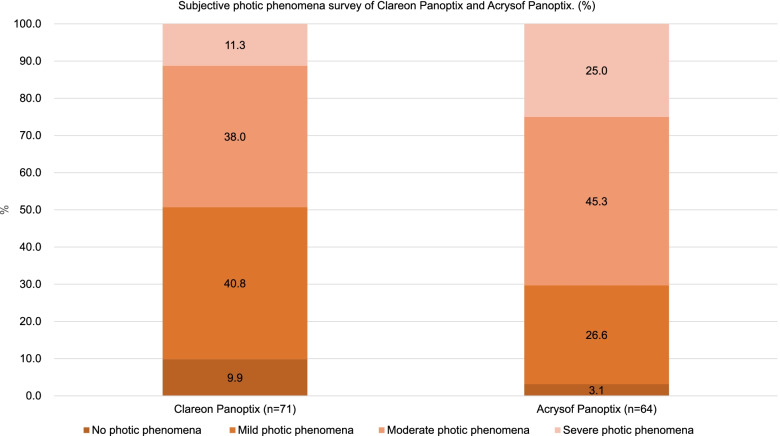
Subjective photic phenomena survey from the Clareon Panoptix and Acrysof Panoptix groups

According to the survey on subjective degree of photic phenomena, 9.9% of CN group patients had no photic phenomena, which was higher (*p* < 0.001) than the TF group (3.1%). In the CN group, mild photic phenomena was the most common (40.8%), while in the TF group, moderate photic phenomena was the most common (45.3%). The incidence of severe photic phenomena was 11.3% in the CN group and 25% in the TF group, which was significantly higher (p < 0.001) in the TF group. (Fig. 3) During the 2-month follow-up period, no complications, such as glistening, surface light scattering, or posterior capsule opacity, were observed.

## Discussion

With the development of multifocal IOLs, cataract surgery is emerging as a conceptual presbyopia surgery that not only removes lens opacity but also corrects refractive errors and provides near vision. This was a breakthrough in the development of diffractive IOLs [1]. TFNT (Acrysof Panoptix, Alcon, Fort Worth, Texas, USA) is the first quadrifocal diffractive C-loop 1-piece IOL and has been widely used for a long time due to its provision of stable distance, intermediate, and near vision, coupled with ease of use [2, 12].

Owing to their optical characteristics, multifocal intraocular lenses have limitations such as dysphotopsia and lowered contrast sensitivity [3]. In particular, IOLs based on Acrysof material may cause glistening or surface scattering in the long term, and multifocal IOLs can be more affected than monofocal IOLs [6, 7]. To resolve this problem, the manufacturer released CNA0T0 (Clareon IOL, Alcon, Fort Worth, Texas, USA), a new material that can increase the clarity of the IOL. In the material composed of the previous phenylethyl acrylate-phenylethyl methacrylate copolymer, PEMA was replaced with 2-HEMA, a hydrophilic polymer [13]. Therefore, the water content was increased to 1.5%, and the clarity of the lens increased. The CNA0T0 IOL improves clarity and solves long-term problems, such as glistening and surface haze [9, 10, 14].

Based on this updated technology, the manufacturer developed CNWT (Clareon Panoptix, Alcon, Fort Worth, Texas, USA), which has the optical structure of TFNT but is made of CNA0T0 material. To the best of our knowledge, this is the first study to report clinical results for multifocal IOL use with this novel material.

To examine the correct optical structure, a defocus curve of corrected visual acuity is required. However, since uncorrected vision is very important to clinical results, a defocus curve for uncorrected visual acuity was collected. Instead, the range of the refractive error was thoroughly managed within 0.5D. The visual acuity results and defocus curve of the CNWT-implanted eye in our study were not markedly different from previously published TFNT defocus curves. This result was considered reasonable because it was based on the same optical structure. The TFNT defocus curve has an M-shaped structure in which good visual acuity is obtained at a distance and then decreases, while the visual acuity increases again at − 2.0D and subsequently decreases [15, 16]. The defocus curve of the CNWT patient in this study also has a similar structure that rises once more at − 2.0D, and shows a smooth visual acuity curve of 20/20 or better at distance and 16/20 or better from 0D to − 2.0D with binocular vision (Fig. 2).

Optical quality indicators using the OQAS did not show a significant difference. However, the average value was better in the CN group, and further research over a longer period of time is required. In the previous study, surface light scattering was less in IOLs with HEMA, and this difference increased as the follow-up period did [9]. As time passes, it is thought that similar results can be obtained with multifocal IOLs; this study was not expected to show a significant difference, due to our limited follow-up period.

The difference in contrast sensitivity between the two groups was most noticeable (Table 2). This result was the most meaningful, and it was more prominent than the existing monofocal IOL study [9]. Although the measuring instrument was different in the previous study, the AULCSF was not statistically significant in either group for up to 7 years after surgery. However, in our study, we found that the CN group performed significantly better than the TF group in the contrast sensitivity test performed 2 months after surgery. This suggests that multifocal IOLs may be more optically sensitive than monofocal IOLs and that the properties of materials with improved clarity can actually make a functional difference. In addition, when the change becomes more pronounced over a long period of time, we considered that the contrast sensitivity result could also have a larger difference over time, but this requires further research.

The subjective degree of photic phenomena was also different between the two groups (Fig. 3). However, it is important to note that we measured the photic phenomena and not the positive dysphotopsia, this is why the percentage with moderate and severe photic phenomena was above 49.3%.

There was no difference in visual acuity, nor posterior capsule opacity or surface haziness. Nevertheless, we believe that the reason for the differences is that the clarity of the material can affect visual function, even without any observation of turbidity on a slit-lamp microscope [3, 8, 17].

Another potential advantage of HEMA-containing material is its mechanical stability. Although not statistically significant, the CNA0T0 IOL showed the least variation when pressure was applied to the long axis of the IOL under the same conditions [18]. This could not be analyzed as data, but during surgery, we realized that the CNWT IOL expanded faster than the prior TFNT IOL and stably settled in the posterior capsule. We believe this could assist in prevention of organic complications. It has already been reported that 100% of the predictive refractive error within 1D can be treated with optimization of the monofocal CNA0T0 IOL [19]; further research is underway for prediction of refractive error.

Although this study was planned prospectively, it was conducted as a retrospective study due to the nature of the hospital, and there were limitations in the follow-up period and number of samples. Due to the short follow-up period, it was not possible to accurately confirm the expected long-term stability and superiority of the new material in future. Therefore, additional follow-up observations and studies are required.

Another limitation was that we included patients with toric IOLs and patients with spherical IOLs together. To avoid confounding factors, it is recommended to use only spherical lenses. However, due to the characteristics of multifocal cataract surgery, many patients had no choice but to use a toric IOL in order to obtain satisfactory visual outcome. The excellent effect of toric multifocal IOLs has already been proven [20], and the authors compared the toric powers of the IOL used to minimize astigmatism variables, and there was no statistically significant difference between the two groups. In addition, to minimize the effect of postoperative astigmatism, strict inclusion criterion within 0.5D was applied.

Nevertheless, this study is the first to report the clinical results of a new multifocal IOL (Clareon Panoptix, CNWT, Alcon, Fort Worth, Texas, USA), a diffractive multifocal hydrophobic IOL made of a new material including HEMA. In addition, it has clinical significance in that it showed a statistically significant difference in contrast sensitivity compared to the existing multifocal IOL (Acrysof Panoptix, TFNT, Alcon, Fort Worth, Texas, USA).

## Conclusions

In conclusion, multifocal IOL including HEMA is a new presbyopia-correction IOL that can be expected to have long-term stability, along with stable optical results. It showed excellent performance similar to that of conventional multifocal IOLs and improved contrast sensitivity. Further studies with longer-term follow-up and higher sample numbers are needed.

## Data Availability

The datasets used and/or analyzed during the current study are available from the corresponding author upon reasonable request.

## Abbreviations

IOL: Intraocular lens
PEMA: Phenylethyl methacrylate
HEMA: Hydroxyethyl methacrylate
UDVA: Uncorrected distance visual acuity
OQAS: Optical quality analysis system
OSI: Objective scatter index
MTF: Modulation transfer function
SR: Strehl ratio
AULCSF: Area under log contrast sensitivity function
LogMAR: Logarithm of the minimum angle of resolution
